# A small-scale study on dancing for people living with Parkinson’s disease

**DOI:** 10.1371/journal.pone.0335433

**Published:** 2025-12-12

**Authors:** Ann Björkdahl, Sofi Fristedt, Katarzyna Filipowicz, Paula Bergman, Ida-Klara Johansson, Iolanda Santos Tavares Silva

**Affiliations:** 1 Sahlgrenska University Hospital, Occupational Therapy and Physiotherapy, Gothenburg, Sweden; 2 University of Gothenburg, Sahlgrenska Academy, Institute of Neuroscience and Physiology, Gothenburg, Sweden; 3 Jönköping University, School of health and welfare, Jönköping Academy for Improvement of Health and Welfare, Jönköping, Sweden; 4 Lund University, Faculty of medicine, Department of Health Sciences, Lund, Sweden; 5 Public Health Department Region Jönköping County, Jönköping, Sweden; Neighborhood Physical Therapy, UNITED STATES OF AMERICA

## Abstract

**Background:**

People with Parkinson’s disease (PD) need life-long rehabilitative interventions to slow disease progression and reduce impact of the disease on daily life activities. To be sustainable for the individual, activities should have a positive impact on physical, cognitive and mental health, and should be appealing and meaningful.

**Aim:**

To quantitatively evaluate the health-related effects of a 10-week dance program for patients with PD.

**Methods:**

A cross-over design with two groups. Group 1 undertook 10 weeks of dance classes; after that, group 2 began their 10 weeks of dance classes. Assessments of both groups at four timepoints (baseline, 10, 20 and 30 weeks) included physical tests and self-reported questionnaires to assess cognition, self-efficacy, well-being, fatigue, and health-related quality of life. The analysis involved comparisons of results after the dance and non-dance periods, for all instruments.

**Results:**

The analyses could not show any significant differences between the assessments after the dance period compared to after the no dance period.

**Conclusion:**

Despite the lack of quantitative evidence of the positive experiences earlier described from focus groups, the study provided insights into how future research could be organised to better capture the multifaceted benefits. Furthermore, the study provides additional evidence that dance as a health-promoting activity in Parkinson’s disease should be viewed in a longer-term perspective.

## Introduction

Parkinson’s disease is a chronic progressive condition affecting physical, cognitive, psychological and social aspects. Due to the combination of motor and non-motor dysfunctions, the consequences of PD may result in a significant limitation of activity. The motor and non-motor symptoms of PD are difficult to manage with current medications; however, exercise has been identified as a possible complimentary treatment that can limit the impact of the disease and improve performance of daily activities [1]. Physical activity or exercise may reduce fatigue and depression, and improve gait, balance and strength and has also shown a positive effect on sleep, memory and quality of life [1,2].

Dancing enables people with PD to participate in an enjoyable form of physical activity within a group. Evidence suggests that dance improves motor impairment, specifically balance and motor symptoms, in individuals with mild to moderate PD [3]. Dance used for therapeutic purposes is both safe and feasible, and has beneficial effects on walking, freezing and health-related quality of life [4]. Dance for PD, a program of dance classes aimed at persons with PD, is an intervention that may be beneficial through a wide range of outcomes [5]. The primary outcome of most randomized controlled studies of Dance for PD is an improvement in motor symptoms, despite non-motor symptoms being the most impactful with respect to quality of life [6]. Apart from motor improvements, dance may increase social and emotional well-being [7] as well as cognitive function [5], and provide a social network coupled with enjoyment of music [8]. Evidence from qualitative studies indicate that a majority of participants experience positive effects with respect to regaining a sense of positive identity, improved physical function, emotional well-being and quality of life, and reduced motor symptoms and feelings of isolation [8].

Different dance forms (including ballet and modern dance styles) have positive effects on PD symptoms and can be tailored specifically to a patient group [9]. Significant improvements have been reported regardless of whether people participated in the dance class in pairs, groups or individually. Improvements are independent of the dance program, dance style, or how long the person has participated in the dance class [10]. Dance for PD^®^ is a multifaceted intervention developed in 2001 as a collaboration between the Brooklyn Parkinson Group and the Mark Morris Dance Group, Brooklyn, USA. Fundamental to the Dance for PD^®^ teaching method is that professional dancers are recruited as movement experts, and their knowledge of balance, movement sequence, rhythm and aesthetic experience is useful for people with PD. During dance classes, the focus is on the aesthetic experience, technique and joy of dance and movement, not on the PD diagnosis itself or its symptoms [11].

The present study was conducted in parallel to a qualitative study [12]. While qualitative enquiry goes some way toward understanding the positive lived experience of those participating in dance classes, it is unclear what may be driving these experiences, or what impact the physical aspects of dance have in generating them [13]. The current study was conducted to address this knowledge gap and by considering dance as a possible complimentary intervention to traditional rehabilitation, and as an intervention that may improve performance in daily activities and quality of life.

### Objectives

To quantitatively evaluate health-related effects of participation in a 10-week program of Dance for PD.

**Research questions**

- Which health related aspects (physical, cognitive, social and psychological) may be influenced by participation in a 10-week “Dance for PD” program?
- Will the outcome, based on questionnaires and physical tests, differ depending on whether measurements were done directly after participation in a dance period or a non-dance period?
- Do eventual positive results remain until follow-up?

## Materials and methods

The study was approved by the Swedish Ethical Review Authority (Dnr: 2020–05822, 2020-11-30) and complied with the Declaration of Helsinki. All participants made a written consent to participate.

### Design and context

**Fig 1:**
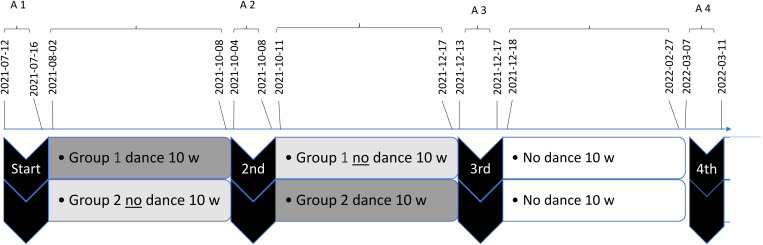
Illustration of the cross-over design with dates for the four assessments (A1-A4), with group 1 dancing during the first 10 w period and group 2 dancing during the second 10 w period and thereafter a follow-up period. The assessments included physical tests and an online questionnaire with five instruments performed at each assessment.

The study has a cross-over design, with two groups participating in the dance intervention for 10 weeks each, starting with the first group dancing, and followed by the second group 10 weeks later. Baseline assessments for both groups were made at the start of the study (A1), a second assessment after 10 weeks (when group one had completed the intervention) (A2), a third after another 10 weeks (when the second group had completed the intervention) (A3), and the fourth and last assessment was made at a follow-up conducted 10 weeks after the second group finished the program (A4) (Fig 1).

### Participants

**Fig 2:**
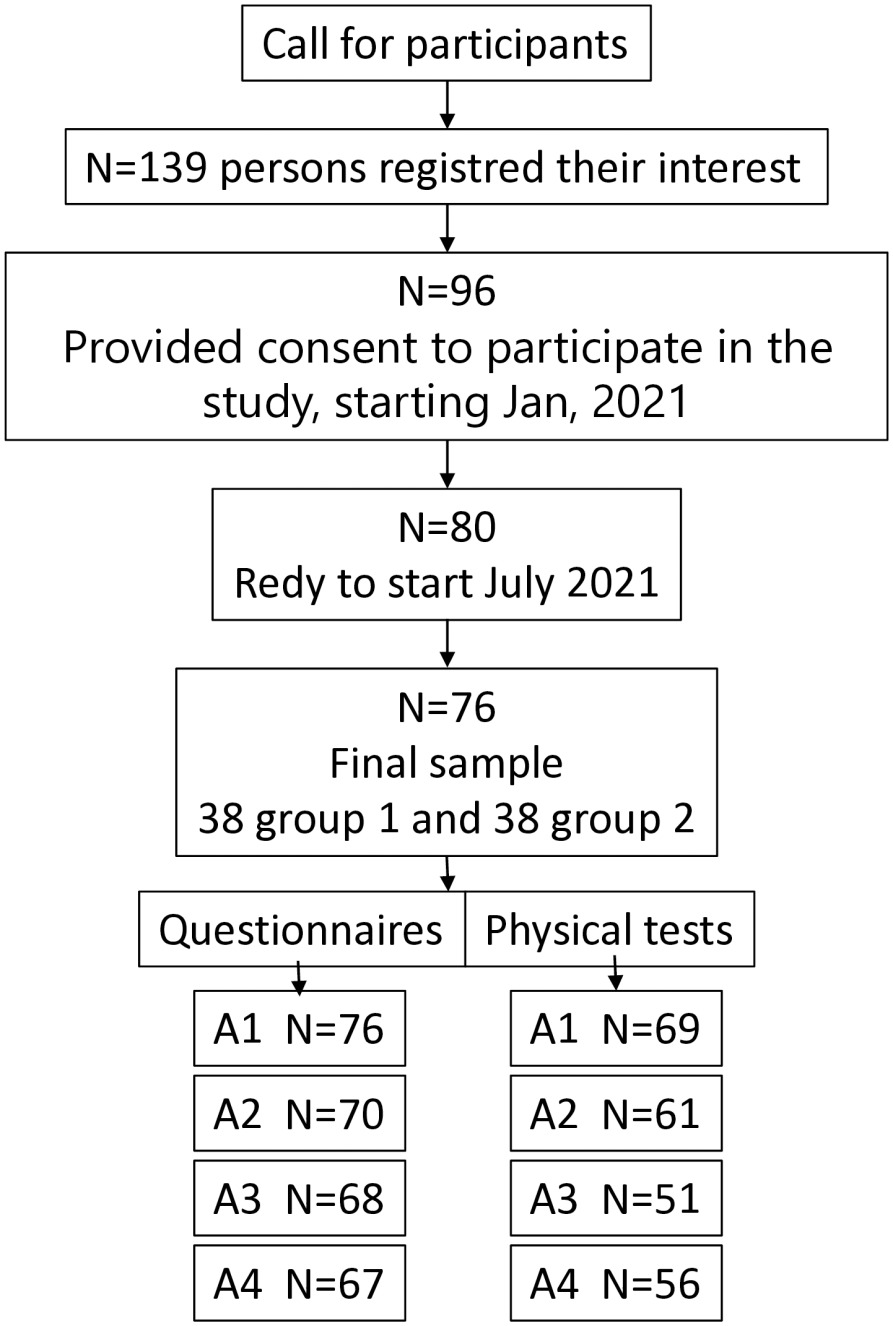
Flow-chart showing the number of participants included in the study and the number assessed at each assessment.

A request to participate in a research study was sent out to dance instructors in Swedish dance networks including the Dance for PD network and researchers in the area, in Sweden. Positive responses were received from dance instructors in 12 cities and from researchers in two cities. Participant recruitment for the study was conducted in the community and included persons who chose to participate in dance classes as part of their everyday life. Recruitment to the study was done during the Autumn of 2020 through contact with PD associations, advertisements in the PD journal and through contact with health care clinics. When registration of interest closed in December, 2020, 137 persons with PD were registered (Fig 2). The study was planned to start in January 2021 but was postponed until August of the same year due to the COVID-19 pandemic. For some who had expressed interest in participating in the study, things had changed during that time, and they withdrew their registration due, for example, to health-related problems, difficulties with transportation and the class time not fitting their schedule. Since some of the locations could not recruit enough participants (≥ 6 persons/group), they had to be removed from the study. Finally, the study started in July 2021 and enrolled 80 participants (all of whom provided informed consent). The final sample consisted of seven locations and a total of 76 participants (four of the included participants changed their mind after providing consent). The sample was divided into two groups where group 1 (N = 38) started with the dance intervention, and group 2 (N = 38) waited for 10 weeks before starting their 10-week dance intervention. Due to the relatively low number of participants, it was not possible to have two groups at all locations; therefore, three locations with too few participants were redistributed into either group 1 (two locations N = 15) or group 2 (one location N = 10).

There were some missing data, as shown in Fig 2. The reason for non-responses to the questionnaires may include difficulties in completing them, fatigue, or simply that they were forgotten. Efforts were made to get as many responses as possible, including the sending of several reminders and the offer to provide paper questionnaires with reply envelopes to those who preferred this from digital questionnaires. Some data were missing from physical tests, due to participants not turning up for an appointment, sickness or difficulty in performing the tests due to a bad period of the disease. Participants who had not been assessed were contacted and arrangements were made to make a new appointment. Since the participants were their own controls and the comparison was not about comparing groups but different conditions, i.e., with or without dance, it was decided not to compensate for dropouts using any intention-to-treat strategy.

Because dance can be adapted to suit the abilities of the different participants, no exclusions were made due to disability. At the start, the participants self-reported their functional level based on Hoehn and Yahr [14], which was used as a basis for ensuring equivalence between and within groups at different locations. At the start of recruitment, the question arose as to whether it was possible for persons who had participated in Dance for PD before to be part of the study. As we did not wish to unreasonably discourage or exclude potential participants, it was decided that previous participation was acceptable on the condition that participation in any form of dance (other than the study itself) was stopped 10 weeks before the study began, and not started again until the 30-week follow-up was completed.

### Dance intervention

The intervention involved 1 hour of dance per week for 10 consecutive weeks. The leader of the dance class was a dance instructor trained in the concept Dance for PD^®^. A training course for the dance instructors was held before the start of the study. All dance instructors were accompanied by an assistant, whose role was to be able to step in if any element of the class required adaptation, or if something occurred in the group; this enabled the dance instructor to focus on leading the dance class. Participants dependent on more continuous help from another person had to arrange support from next-of-kin or bring a personal assistant. Participants also needed to be able to get to and from the dance venue on their own.

The program was based on the “Dance for PD® concept” developed by the Mark Morris Dance Group in collaboration with the Brooklyn Parkinson Group, and on previous research in the field [11]. Each session contains the following six main elements, which have the same purpose each time; however, the level of difficulty increases gradually over the course of the program.

1. Warm-up sitting on chairs to prepare the body, respiratory system and brain; to increase blood circulation; to increase awareness of the whole body and other dancers in the room (including the leader).
2. Ballet barre or chairback exercises with different dance styles and music to connect mental and physical aspects, and to practice different movement qualities.
3. Transfer exercise aimed to help participants increase self-confidence with respect to balance and movement without support/with support. Walking exercises that focus on the feet and on rhythm, aimed at getting participants to think about and get to know their walking pattern.
4. Choreography (in the middle of the lesson) focusing on the joy of movement through longer and varied dance sequences. It can be compared to a mental puzzle and aims to affect non-motor symptoms such as slowness/delayed thought process, memory difficulties, and coordination of the right and left halves of the body.
5. Improvisation/pairing exercises that promote creativity and imagination but also provide social interaction and touch.
6. Cooling down/relaxation focusing on your own body and yourself.

### Evaluations

The evaluations included two parts: questionnaires and physical tests. The questionnaires were sent out for each of the four assessments and answered online or in paper form; At each assessment, the same physical tests were carried out by the same licensed physiotherapist at a clinic at the actual location. Instructions regarding measurements were drawn up and communicated to the evaluating physiotherapists via an online meeting before the study start. The examiners were blinded to the group membership.

The following tests and questionnaires were included:

*Physical function*: the focus was on balance, walking (endurance), dual-tasks and posture, as measured by the Mini-Balance Evaluation Systems Test (Mini-BESTest), The Timed Up & Go Test (TUG), the Diff-TUG (dual task with TUG and cognitive task and TUG and manual task carrying a glass of water) and the 6 min walking test.

The mini-BESTest is a short version of the BESTest, and contains 14 items from four of the subsystems of the BESTest; it is believed to measure dynamic balance [15]. It has been translated and validated in Swedish [16].

TUG is a test of balance and basic mobility; it is commonly used to examine functional mobility. The test requires a subject to stand up from an armchair, walk 3 meters, turn, walk back, and sit down. Time taken to complete the test correlate strongly with the level of functional mobility [17]. TUG is included in the Mini-BESTest as a TUG dual-task requiring cognitive performance (i.e., mathematical subtraction) while doing the TUG (i.e., the TUGcognitive) [18]. The dual-task TUGmanual (performing the TUG while carrying a glass of water) was also included [19]. Both the dual-tasks (the cognitive and manual TUG) are used to generate a DiffTUG by calculating the difference between the time for the basic TUG and that for the cognitive or manual TUG.

*To capture non-motor, social and emotional problems*, the following questionnaires were used:

The Cognitive Failure Questionnaire (CFQ), which contains 25 questions about cognitive failures that occur in everyday life [20]. The response options range from 0 (never) to 4 (very often). Total scores range from 0–100, with a lower score indicating fewer problems.

The Parkinson Fatigue Scale (PFS-16) [21]. Sixteen questions, each with five response options from 1 (not at all correct) to 5 (completely correct), are asked. The total scores range from 16–80. A lower score indicates less fatigue.

The Psychological General Well-being short (PGWB-S): this asks six questions about anxiety, energy and mood [22]. Answers are from 1 (negative) to 6 (most positive). The total score ranges from 6–36 and is multiplied by 3.66 to allow comparison with the original 22 item version (0–100). A higher score indicates better well-being.

The EQ5D-5L, which measures health-related quality of life: the test comprises five questions about mobility, personal care, activities of daily living, pain and anxiety/distress and a Visual Analogue Scale (VAS) for perceived health (Euroqol, EQ5D-5L) [23]. In the present study only the EQ-VAS component was used, for which scores range from 0 (worst health) to 100 (the best possible health).

The General Self-Efficacy Scale (GSE), which assesses the belief in one’s own ability to cope with a broad range of stressful or challenging demands [24]. It contains 10 questions, each with a score ranging from 1–4: a score of 1 means low belief in competence and a score of 4 means the greatest belief in competence. A total score (range, 10–40) is calculated from all items; a higher score indicates greater self-efficacy.

### Data analysis

The aim of the study was to explore the potential effects of the dance intervention with the cross-over design being divided into three periods under two different conditions: the dance period (ΔA), the no dance period (ΔB), and the follow-up period. The two Δ conditions were used for comparison (Fig 1). Data analysis aimed to find out whether one of the two Δ conditions had a more favourable outcome, i.e., the null hypothesis was that there will be no difference in outcome between condition ΔA (dance) and condition ΔB (no dance).

Descriptive data are presented for the whole sample and for each of the two groups as the mean, standard deviation (SD) and as the min-max sum scores. ΔA represents the sum scores for all instruments calculated immediately after the “dance” period, i.e., at assessment two (A2) for Group 1 and at assessment three (A3) for Group 2. ΔB represents the sum scores for all instruments calculated immediately after the “no dance” period: i.e., at A3 for Group 1 and at A2 for Group 2.

Comparisons between the two conditions (ΔA and ΔB) were made using Student’s t-tests for paired samples to explore whether the results obtained immediately after the “dance period” are significantly better than those after the “no dance” period. Significance was set to p < 0.05. Paired t-test of sum scores at the start (i.e., A1) compared with those at the 10-week follow-up (i.e., A4) were also made to explore persistence of eventual benefits of the dance intervention, for the whole sample.

To examine the importance of the order of the dance intervention (dance first or dance after waiting for a 10-week period), a standard multiple regression analysis was performed, with each of the different instruments entered in the model as a dependent variable, and time as an independent variable. The dependent variables comprised the difference between the two conditions (ΔA-ΔB). The independent variable, time, was dichotomous with dancing in the first period or dancing the second period; the aim was to find out if the order matters.

## Results

The final sample comprised 76 participants. Table 1 illustrates how the cohort was divided into the two groups. The two groups were similar with respect to age, and both groups contained more women (69% and 59%). More of the participants in group 1 had experienced Dance for PD before. The time from a diagnosis of PD varied a lot, from 3 to 23 years. Around 50% in both groups received a diagnosis less than 7 years ago. The Hoen & Yahr ratings showed that approximately 40% of the sample were in an early phase of disease, and still had good balance and mobility. The largest proportion of the sample had a Hoen &Yahr rating of III, meaning that they had problems with balance but remained physically independent. From the Hoen &Yahr rating, one may conclude that both groups had similar levels of function. The dance instructors kept attendance lists during the period of the classes, and most participants were motivated and tried to attend at all sessions; however, due to the nature of their condition, a few participants failed to attend all 10 sessions, mainly due to health-related problems, falls, or operations. Attendance ranged from 5–10 sessions, with the most common number being ≥ 8.

**Table 1:** Description of the study participants.

	Total sample N = 76	Group 1 N = 38	Group 2 N = 38
Age, mean (SD, min-max)	69.26 (8.91,42-85)	70.56 (8.51, 49–84)	67.95 (9.21, 42–85)
Mean time with PD (years; SD, min-max)	9.51 (5,65, 3-33)	9.77 (5.61, 3–23)	9.27 (5.76, 3–32)
Gender male/female	35%/65%	31%/69%	41%/59%
Hoen & Yahr I	24.5%	19.2%	30.4%
Hoen & Yahr II	16.3%	19.2%	13.0%
Hoen & Yahr III	46,9%	46.2%	47.8%
Hoen & Yahr IV	12.2%	15.4%	8.7%
Earlier participation in Dance for PD yes/no	42.7%/57,3%	49%/51%	37%/63%

Table 2 presents data from both two groups obtained from the physical tests and questionnaires. At baseline, the first assessment (A1), there were no significant differences between the groups with respect to any of the instruments. Changes in the scores on all instruments for all assessments were small.

**Table 2:** Descriptive of the scoring of the questionnaires and physical tests at the four assessments in each of the two groups (1 and 2).

	Group 1				Group 2			
Instrument	N	Mean	SD	Min–max	N	Mean	SD	Min–max
PFS A1	38	46.82	14.88	16–68	38	50.08	15.29	18–80
PFS A2	38	46.79	12.48	21–70	32	46.50	14.49	16–80
PFS A3	37	47.97	14.50	16–74	31	47.71	17.62	16–80
PFS A4	35	46.14	16.36	16–75	32	49.06	15.63	16–76
GSE A1	38	27.82	5.53	16–-37	38	27.95	4.84	19–36
GSE A2	38	27.45	5.75	17–38	33	27.42	5.62	18–38
GSE A3	37	28.14	5.33	19–39	31	27.26	6.23	13–39
GSE A4	35	27.14	4.88	16–37	32	28.66	6.58	12–40
PGWB A1	38	68.77	21.12	26–99	38	62.22	17.41	26–95
PGWB A2	38	66.65	17.50	22–102	33	59.89	22.66	0–106
PGWB A3	37	65.98	20.33	22–99	31	63.52	23.04	15–106
PGWB A4	35	64.10	24.05	15–106	32	65.77	18.53	29–95
CFQ A1	36	56.00	13.92	33–91	37	57.92	14.01	29–97
CFQ A2	37	58.27	15.44	34–91	32	56.19	13.94	27–84
CFQ A3	37	58.73	15.45	33–93	31	57.06	14.31	30–88
CFQ A4	35	58.89	14.93	36–92	32	59.31	16.58	28–93
EQ5D A1	37	59.46	16.60	30–87	33	54.58	19.42	1–84
EQ5D A2	37	63.27	19.73	20–90	29	57.76	20.39	8–87
EQ5D A3	32	55.31	18.82	23–88	28	56.71	25.04	5–91
EQ5D A4	30	53.90	20.88	15–84	27	56.78	17.75	20–1
6MWT A1	31	408	127	69–630	31	408	128	35–585
6MWT A2	34	416	117	66–622	27	439	105	245–600
6MWT A3	28	438	94	210–615	23	457	107	242–657
6MWT A4	30	445	105	154–656	25	476	94	312–675
M-B A1	36	19.08	5.81	1–26	33	19.21	5.54	7–28
M-B A2	34	20.32	4.51	9–27	27	20.78	4.93	6–28
M-B A3	28	21.61	4.52	9–27	23	23.17	3.14	17–27
M-B A4	31	22.19	4.91	8–28	25	23.00	3.73	14–28
TUG A1*	33	11.55	4.06	7–29	34	11.35	4.86	6–28
TUG A2*	32	11.36	4.58	7–29	27	10.57	3.55	5–19
TUG A3*	28	12.43	10.06	7–60	23	10.07	3.30	5–18
TUG A4*	29	10.36	3.71	7–25	25	9.04	1.83	6–13
TUGcog A1*	33	16..07	5.41	9–28	34	17.52	10.03	7–56
TUGcog A2*	30	16.65	6.90	8–34	26	14.73	7.43	5–40
TUGcog A3*	28	17.29	12.99	8–77	23	15.17	5.97	6–32
TUGcog A4*	29	14.59	5.11	8–28	25	13.20	4.14	6–24
TUGglas A1*	33	13.72	5.25	7–36	34	13.29	8.65	7–55
TUGglas A2*	31	13.54	5.97	8–42	25	12.68	4.78	7–25
TUGglas A3*	28	15.54	13.44	8–77	23	11.91	4.32	6–23
TUGglas A4*	29	12.56	5.28	8–37	25	11.00	2.32	7–15

We found no significant differences between the conditions of dance and no dance (ΔA and ΔB) based on questionnaires (PFS, GSE, PGWB-S, CFQ, and EQ5D-5L VAS) or the physical tests (6 MWT, miniBest, TUG, and TUG manual and cognitive). Although there were no significant differences between the conditions, and few changes in the outcome of the different assessments, regression analysis was conducted to explore any effects of the order of dance participation on outcome. Preliminary analyses were conducted to ensure no violation of the assumptions needed for the analysis. The models identified no, or very little, variance in the dependent variable, suggesting that order was not a factor. Adjusted R squared values were 0.0001–0.019, and the standardized Beta values were 0.025–0.390.

There were no significant differences in the data of all participants obtained at the start (A1) compared to follow-up (A4) 30 weeks later.

## Discussion

The study found no significant differences between the dance/no dance condition with respect to health-related outcomes, such as physical, cognitive, self-efficacy, fatigue, well-being or perceived health, after participation in “Dance for PD”. The null hypothesis is maintained. This is in line with a previous dance study that found insufficient evidence to warrant development of specific guidelines; that review concluded that apart from a modest improvement in balance, there was insufficient evidence of improvements in motor and cognitive function, agility, mood, social outcomes or quality of life [10]. However, given the progressive nature of the disease, the results can perhaps be interpreted favourably as there was no significant deterioration from baseline to 30 weeks. A present study with long-term follow-up over 3 years found that dance can reduce the rate of deterioration [9].

A reason for the lack of evidence may be the use of different methodologies; one that looks at clinical signs as the primary outcome and another that places emphasis on what dance can offer participants in a more holistic way [25]: using the latter makes it more difficult to capture data quantitatively. For instance, how can a generic measure capture experiences such as ‘moving more freely’, ‘feeling looser’, ‘feeling less isolated’ or ‘escaping the condition’ [13]. The current literature highlights some of the multidimensional qualities of Dance for PD based on a variety of measures; however, these are universal measures that do not allow for appreciation of the diverse effects that dance may have on an individual. For some, dance improves physical symptoms, but for others, the connection with others in the room and the emotional feeling are more important [13]. The present study hoped to capture both aspects, but the results indicate that the design and instruments were not optimal. McGill et al. [25] emphasize the use of the ICF model as an explanatory frame for research that aims to examine not only how dance may affect clinical signs and symptoms, but also to understand how dance may affect participation in daily activities and life experiences. Hulbert et.al 2020 [13] made an interesting attempt to explore the effects of dance through convergent parallel mixed method combining measurements of whole-body movement with the lived experience and found it feasible to map the experience of dance (what I feel) against biomechanical change (what you see) following dance.

The study has several limitations. First, the sample size was relatively small, although we tried to broaden the inclusion criteria as much as possible. The number of people with PD in a small to medium-sized city, who are aware of the possibility of, and are interested in participating in, a dance study was limited; however, living in a big city can mean a longer travel distance/time from home to the dance studio, which may be a reason for withdrawal once this becomes apparent. Other means of informing potential participants about the study might also have been more successful. The need to postpone the start of the study due to the pandemic also reduced the sample size. The plan was to start the study in the spring because that period allows more time (in weeks) than the fall before there is a need to take a break; however, the delay meant that the study started in July with the first assessment. This introduced other problems in that many people take holidays at this time of year. Another limitation was the participation in the dance classes which, due to the nature of the disease and other injuries or illnesses, meant that some participants could not attend all 10 sessions. As the number of sessions was limited from the outset, it is possible that absences on individual occasions may also be significant for the result.

Running the study at different locations to get a large sample made it difficult to ensure that the conditions for the interventions were as equal as possible at all locations. Therefore, this was addressed through training those dance instructors who did not already have training in the concept of Dance for PD. Standardization regarding location and setup was also ensured. Measurement instructions were drawn up and communicated to the evaluating physiotherapists via an online meeting; however, factors such as the dance instructors’ experience, engagement, and personality, all of which might have affected the outcome and participant’s expectations, could not be controlled.

The instruments used in the study are well-known and validated, and most have been used in similar studies. To be able to evaluate aspects such as self-efficacy, wellbeing and experience of health, the instruments needed to be based on self-ratings by the participants. The questionnaires address issues that are quite comprehensive and can be influenced by a great many different factors; thus, they may not be sensitive enough to identify small differences that may occur within the relative short period of the dance intervention. Sleep difficulties, failure to perform something important, or just having a cold can give rise to a worse assessment of fatigue, coping skills, health and well-being [26]. Due to the ceiling effect noted in other studies, the fact that this was not de novo study may have also contributed to the outcome. However, it was not possible to compare those who had danced before the study with those who had not, as such a comparison would not be accurate due to significant individual differences in the severity of Parkinson’s disease, which were not evenly distributed between those who had danced and those who had not in the sample. It would have been useful if personal contextual factors were taken into consideration because such things can bias the result. The nature of the disease and its fluctuations can create difficulties for participants, making it difficult to know how to answer the questions, thereby introducing a high degree of variability into the data [27].

Performing clinical studies in an environment that is not controlled is challenging. The participants were ordinary people with PD and living in a community setting. Even though they all have PD, they may be very heterogeneous in terms of lifestyle, social conditions, and interests; thus, they may have entered the study with very different expectations. The large variance in all outcome variables suggests that such differences between the participants in the study may have influenced the result.

The literature suggests that the duration of interventions varies between short periods of 2–3 weeks, medium periods of 10–13 weeks, or longer durations of 6–12 months, and that the number of classes per week ranges from one to three [28]. The present study program scheduled one dance class per week and was of medium length (i.e., 10 weeks). Earlier studies have reported positive effects of dance [3,4]; however, further research is needed to determine which program design (i.e., frequency and duration) as well as which program elements are the most important to bring about positive, clinically meaningful changes in people with PD [3,28]. Most studies reporting a positive effect provided dance classes twice a week. Based on the literature, it seems that the best balance is a 1-hour dance class, twice a week, for a period of 10–13 weeks [5,28,29]. One way to increase the intensity could be to follow the example of Rocha et al. [30] who combined on-site dancing with a home programme. [28]. Despite the great impact of non-motor symptoms, the primary outcome of trials often focuses on motor symptoms. The greatest effects on motor impairment have been achieved after dance classes given over a longer duration [28]. A higher “dose” of dance activity may also increase quality of life through increased social participation. A more intense program with a longer duration may have improved the results of the present study. However, one of the main goals of dance as an activity in the context of Parkinson’s is to find activities that promote health in the long term and may need to be less intensive than the goal of rapidly improving physical function [2].

Dance is a physical activity that can be used for both personal pleasure and to improve one’s social life; it is a means to improve health within a multidimensional, enriched environment that provides the tools to enhance flexibility, creativity, cognitive and physical functions, and well-being [9]. Compared with exercise, dance appears to have a stronger effect on balance, but not on other functional outcomes [31]. Comparing dance with matched exercise interventions also yields different outcomes due to the more artistic elements, which may affect emotional responses, the experience of beauty, self-efficacy, and gait performance [6,9]. The music provides a rhythmic cue, which appears to improve gait and stride length, and may reduce the risk of falling [32,33]. The outcomes of dance are important for functioning in daily life, and for well-being. Dance for PD studies show high compliance, few drop-outs, and a high percentage of continued activity after the study period [30,31], all of which are important aspects with respect to the life-long need to undertake preventive activities and reduce the sequalae of the disease.

The present study focused on non-motor symptoms as well as on psychological and social aspects, as did previous research reports, but we failed to show improvements using this study design. Similar to the present study, Houston et.al. [27] did not find any improvement in outcomes and suggested that a longer duration of the intervention could have made a difference. A qualitative study based on the present cohort found that the desire to continue dancing, as well as a hesitation to give up dancing, was a motivation for those who had danced before [12]. This indicates that dance is a physical activity that can be continued long-term, which is a great advantage because PD is a chronic progressive disease. These results are supported by Bearss et. al. [9], who reported the positive benefits of longitudinal weekly training for slowing progression of the motor and non-motor symptoms of PD. The holistic approach of dance means that it not only has health-related positive effects on quality of life, by improving mood and decreasing clinical symptoms of anxiety and depression, but it is also enjoyable and socially engaging [13]. This seems to increase motivation to start and maintain physical activity and improve cognition, thereby facilitating management of this long-term condition [13]. It has been suggested that dance can directly enhance well-being through its neurological, social, and psychological benefits, while the physical benefits indirectly influence well-being, thus forming a positive loop that encourages continued participation in dance [34]. A systematic review from 2024 of the literature on the effectiveness of structured dance interventions, compared with structured exercise programs, on psychological and cognitive outcomes across the lifespan found preliminary evidence that dance could be superior to other physical activity interventions to improve motivation, aspects of memory, and social cognition, distress and could also improve stress, self-efficacy and language fluency [35].

The qualitative study from the same cohort as the present described the participants multifaceted experiences from the dance in four sub-themes: “*Connectedness*, *Pleasure and glamour*, *Well-being in mind and body* and *Customized movements”*, that all contributed to the benefits of Dance for PD [12].

Although we could not prove the benefit of dance for PD in the current study, there is extensive support in the literature, as discussed in the article, to suggest dance as an ongoing health-promoting activity. Patients are not looking for large short-term effects; rather they want to see smaller, long-term and health-promoting effects. Against this background, we believe that evaluation of the benefits of dance should also be carried out over the long term, and above all using tools that can capture the importance of dance in terms of slowing progression and improving well-being and overall health. Future research should therefore focus on designing holistic research evaluating the long-term benefits of Dance for PD. To quote another study, we should “move beyond the questions of “does dance work” to understanding what it is about dance that makes it work for each individual and demonstrate that multiple models are at play in the dancing experience” [13].

## Conclusions

With the current study design and the instruments included, the study was not able to quantitatively demonstrate the multifaceted positive experiences previously presented from interviews with the same participants [12]. A possible conclusion of this may be that the benefits of dance are individual and vary. Larger samples, higher adherence rates, a de novo study design to eliminate any ceiling effect, and a longer study period could provide better conditions to quantitatively capturing the multifaceted potential benefits of dance in Parkinson’s disease in future research. As proposed by Hulbert et.al. 2020 [13] future research should find ways to demonstrate that multiple models are at play in the dancing experience. Since dance is a physical activity that can appeal to many people with Parkinson’s disease, it should be seen as a complement to other rehabilitation programs with the potential for long-term benefits and is therefore suitable for promoting health in Parkinson’s disease.
